# Mesenchymal stem cells decrease lung inflammation during sepsis, acting through inhibition of the MAPK pathway

**DOI:** 10.1186/s13287-017-0734-8

**Published:** 2017-12-22

**Authors:** Leonardo Pedrazza, Monica Cubillos-Rojas, Fernanda Cristina de Mesquita, Carolina Luft, Aline Andrea Cunha, Jose Luis Rosa, Jarbas Rodrigues de Oliveira

**Affiliations:** 1 Laboratório de Pesquisa em Biofísica Celular e Inflamação, Pontifícia Universidade Católica do Rio Grande do Sul (PUCRS), Porto Alegre, Rio Grande do Sul CEP 90619-900 Brazil; 2 Departament de Ciències Fisiològiques, IDIBELL, Campus de Bellvitge, Universitat de Barcelona, L’Hospitalet de Llobregat, E-08907 Barcelona, Spain; 3 Laboratory of Pediatric Respirology, Infant Center, Institute of Biomedical Research (IPB), Pontifícia Universidade Católica do Rio Grande do Sul (PUCRS), Porto Alegre, Rio Grande do Sul CEP 90619-900 Brazil

**Keywords:** Sepsis, Mitogen-activated protein kinases, Mesenchymal stem cells, Macrophages

## Abstract

**Background:**

Sepsis is a severe medical condition that ranks among the top 10 causes of death worldwide and which has permanently high incidence rates. Mesenchymal stem cells (MSCs) have been found to be potent modulators of immune responses. More importantly, there is evidence that MSCs have a beneficial effect on preclinical models of polymicrobial sepsis. However, the changes caused by the MSCs in the effector cells of the host immune system remain unclear.

**Methods:**

A mouse model of sepsis (male C57BL/6 mice) with three experimental groups was used for experiments in vivo: a control group, an untreated septic group, and a septic group treated with MSCs. In vitro experiments were performed using a cell line of pulmonary macrophages (RAW 264.7) co-cultured with MSCs and stimulated with lipopolysaccharide (LPS).

**Results:**

In vivo we demonstrated that treatment with MSCs was able to reduce the expression of cyclooxygenase-2 (COX-2) and nuclear factor kappa B (NF-κB), and thereby decrease the production of inflammatory cytokines. In vitro experiments using a co-culture of macrophages with MSCs showed a decrease in COX-2 and NF-κB, and showed that this reduction was directly related to the ability of MSCs to inhibit phosphorylation of ERK, RSK, and p38, enzymes that belong to the family of mitogen-activated protein kinases (MAPKs).

**Conclusions:**

This study demonstrated that MSCs are able to inhibit the MAPK pathway activation, modulating the inflammatory response during sepsis. This understanding that MSCs can remodel the response of host cells and improve the course of sepsis is essential for developing new treatments for this pathology.

## Background

Sepsis is a severe medical condition that ranks among the top 10 causes of death worldwide and which has permanently high incidence rates [1]. Even with appropriate antibiotic and resuscitative therapies, sepsis carries a 30% mortality rate and significant morbidity associated with organ failure [2]. Furthermore, it incurs a staggering $16.7 billion cost in the US health economy, with over 750,000 annual cases and greater than 200,000 deaths each year [3].

Sepsis is caused by an infection and involves a complex interaction between the pathogen and the host immune cells, characterized by a systemic inflammatory state [4]. Moreover, the role of the immune response is crucial to fight infection; it is also responsible for the inflammatory tissue infiltration and severe organ damage, both hallmarks of sepsis [5]. Evidence suggests that modulation of pro- and anti-inflammatory factors contributes to the suppression of immune effector cells, induces the systemic inflammation, and causes tissue damage during the sepsis [6].

Over the last few years, many studies have been conducted in order to decrease the mortality rate associated with sepsis, but the pharmaceutical research community is not getting any substantial new messages regarding drug design, development, and therapy [7]. However, as experimental studies have demonstrated in the setting of sepsis syndrome, mesenchymal stem cell (MSC) treatment notably alleviated the sepsis-induced inflammatory reaction, decreased mortality, and improved prognostic outcome [8–12].

MSCs are a subpopulation of multipotent cells that may be isolated from various adult tissues and organs. Several studies have described MSCs as a novel therapeutic strategy for the treatment of diseases related to inflammation and tissue injury because they are potent modulators of immune system, with the ability to regulate both the innate and adaptive immune response [13, 14]. Several studies demonstrate that the protective role of MSCs in sepsis may be attributed to the soluble paracrine factors released by these cells, such as interleukin (IL)-10, prostaglandin E2 (PGE2), tumor necrosis factor (TNF)-α and IL-6 [15–17].

Previous studies in experimental model of sepsis demonstrated the ability of MSCs to reprogram macrophages from a pro-inflammatory state to an anti-inflammatory state through the release of PGE2, causing increased secretion of IL-10. Nevertheless, the pathways that are altered in this reprogramming of effector cells of the immune system are unclear [18].

We know that the cyclooxygenase-2 (COX-2) is responsible for the production of a huge amount of PGE2, which is highly expressed during the inflammatory process [19]. The pathway that usually regulates COX-2 expression in inflammation involves a family of highly conserved intracellular signaling molecules, the mitogen-activated protein kinases (MAPKs) [20]. The MAPK pathway has been implicated in processes regulating cell growth, differentiation, apoptosis, and inflammation. In mammals, there are three MAPK subgroups, the extracellular signal-regulated kinase (ERK), c-JUN N-terminal kinases (JNK) and p38 [21].

Therefore, in this study we investigated the ability of MSCs to decrease COX-2 production and other inflammatory mediators through inhibition of the MAPK pathway. For this purpose we used a model of sepsis previously developed by our laboratory, consisting of the introduction of a sterile gelatin capsule in the peritoneal cavity containing *Escherichia coli* suspension and non-sterile fecal content, subsequently followed by an in vitro investigation using a cell line of lung macrophages, which are important effector cells of the immune system involved in many immunological reactions.

## Methods

### Animals

Male C57BL/6 mice (8–12 weeks old) were kept on shelves in ventilated cages that provide 60 air cycles per hour, with relative humidity ranging between 55 and 65%, a 12-h light–dark cycle, a temperature of 22 ± 2 °C, and free access to food and water. The animals were maintained in accordance with the Guiding Principles in the Care and Use of Animals approved by the Council of the American Physiological Society. The experimental protocol was approved by the Ethics Research Committee of Pontifícia Universidade Católica do Rio Grande do Sul (protocol number 14/00403).

### MSC culture and characterization

Male C57BL/6 mice (8–12 weeks old) were MSC donors. Under sterile conditions, the animals were anesthetized (pentobarbital 50 mg/kg intraperitoneally) and, after the collection of the adipose tissue, mice were killed by cervical dislocation. In humans, adipose tissue is usually collected by needle biopsy or liposuction aspiration; for this reason, we chose to perform the collection of adipose tissue from anesthetized animals and proceed to euthanasia later. This step makes the work closer to what happens in the clinic. Adipose tissue was obtained from the epididymal adipose tissue, cut into small pieces, collagenase digested, filtered, and then cultured using Dulbecco’s modified Eagle’s medium (DMEM; Invitrogen Corporation, CA, USA) without ribonucleosides or deoxyribonucleosides containing 2 mM l-glutamine and 20% fetal bovine serum (FBS; Invitrogen, Carlsbad, USA), with 1% penicillin–streptomycin. Cells were passaged every 3–4 days by trypsinization when they reached 70–80% confluence and were used for the experiments between passages 3 and 4. Between each passage, cell viability was measured using the trypan blue exclusion test. MSCs were cultured in a humidified incubator at 5% CO_2_ and 37 °C under sterile conditions. Before each experiment, cells were trypsinized, counted, washed twice with phosphate-buffered saline (PBS) and resuspended in PBS. MSCs were characterized by expression of the cellular markers (CD90^+^, CD105^+^, CD34^–^, and CD45^–^), determined by flow cytometry analysis (Bio-Rad, Hercules, USA). MSCs were induced to differentiate into adipocytes, osteocytes, and chondrocytes using cell differentiation kits from R&D Systems (Minneapolis, MN, USA) in accordance with the recommendations of the manufacturer.

### Experimental sepsis induction and treatment

The animals were weighed and then anesthetized with a mixture of ketamine (80 mg/kg) and xylazine (20 mg/kg) intraperitoneally. The abdomen of each animal was shaved and cleaned with povidone–iodine solution. A 1-cm midline abdominal incision was made to expose the linea alba. The peritoneum was opened by blunt dissection. Sepsis was induced by introducing a sterile gelatin capsule size “1” in the peritoneal cavity containing another sterile capsule size “2” with the *Escherichia coli* (3 μL; ATCC 25922) suspension and non-sterile fecal content (20 mg). This experimental model was developed by our laboratory [11, 22]. The animals were then divided into three groups: 1) sham (mice were implanted with an empty capsule and received a retro-orbital injection of 100 μL PBS); 2) sepsis (sepsis-induced and received a retro-orbital injection of 100 μL PBS); and 3) sepsis + MSCs (sepsis-induced and treated with 1 × 10^6^ MSCs in a retro-orbital injection of 100 μL PBS at the time of induction). We also used another model of sepsis induced by the injection of lipopolysaccharide (LPS) from *E. coli 026:B6* (12.5 mg/kg; Sigma-Aldrich, St. Louis, USA) intraperitoneally. The animals were divided into the same groups as in the other model. Blood samples were collected using cardiac puncture 12 h after sepsis induction. This time was determined from previous studies in our laboratory.

### Histological analysis

The superior lobe of the right lung was ligated and prepared for histological and morphological analysis. Lungs were perfused with 10% buffered formalin on a gravity column (20 mmHg). Tissue specimens were embedded in paraffin blocks, cut into 4-μm sections, stained with hematoxylin and eosin (H&E), and examined by light microscopy. Other sections were sequentially subjected to immunostaining analysis. After deparaffinization, the sections were sequentially treated with 1% H_2_O_2_ for 10 min and rinsed thoroughly with PBS. Sections were blocked with 2% normal blocking serum in PBS at room temperature for 60 min to suppress any nonspecific binding of IgG, followed by incubation with anti-COX2 (dilution 1:200; Merck Millipore, Darmstadt, GER), or nuclear factor kappa B (NF-κB) p65 (dilution 1:200; Cell signaling, Danvers, USA). The cellular nuclei were stained using TO-PRO®3 (dilution 1:1000; Thermo Fisher Scientific, Waltham, USA). All of the slides were evaluated by confocal immunofluorescence microscopy. We calculated the inflammatory lung tissue score using the software Image-Pro Plus (Medical Cybernetics). The score for peribronchial and perivascular inflammation was evaluated on a subjective scale of 0–3, as described elsewhere [23]. A value of 0 was assigned when no inflammation was detectable, a value of 1 was adjudged for occasional cuffing with inflammatory cells, a value of 2 when most bronchi or vessels were surrounded by a thin layer (one to five cells thick) of inflammatory cells, and a value of 3 was given when most bronchi or vessels were surrounded by a thick layer (more than five cells thick) of inflammatory cells.

### Cytokine quantification

To determine cytokine levels, serum samples were collected from mice 12 h after sepsis induction. Multiple soluble cytokines (IL-6, TNF-α, IL-10) were simultaneously measured using a Luminex Multiplex Assay kit (Thermo Fisher Scientific). We used a luminometer Luminex® 100/200 (Luminex Corporation, Austin, USA) and the results were analyzed using the software xPONENT® Solutions software (Luminex Corporation).

### Macrophage stimulation and macrophage–mesenchymal stem cell co-culture experiments

The RAW 264.7 macrophage cell line was obtained from Sigma-Aldrich (USA) and grown to confluence in DMEM containing l-glutamine (2 mM), penicillin (100 IU/mL), streptomycin (100 mg/mL), and 10% FBS at 37 °C in a 5% CO_2_ humidified incubator. Macrophages were plated in six-well plates at a concentration of 3 × 10^5^ cells in 2 mL per well. After this, MSCs were also co-cultured in a proportion 1:10 with the macrophages in the same well. After 24 h, macrophages and macrophages co-cultured with MSCs were stimulated with LPS from *E. coli 026:B6* (1 μg/mL; Sigma-Aldrich) at different times.

### Western blot analysis

Macrophages or lung tissue were lysed in CHAPS lysis buffer (10 mM Tris–HCl, pH 7.5, 100 mM NaCl, 0.3% CHAPS, 50 mM NaF, 1 mM sodium vanadate, 1 mM phenylmethylsulfonyl fluoride, 5 μg/mL leupeptin, 5 μg/mL aprotinin, 1 μg/mL pepstatin A, 50 mM β- glycerophosphate, 100 μg/mL benzamidine) for 30 min at 4 °C and equal amounts of proteins were separated by electrophoresis. We used Tris-Acetate PAGE systems as previously described [24, 25]. After running the gel, the proteins were transferred to PVDF membranes and viewed by immunoblotting, as described elsewhere [23, 24]. Band intensities were analyzed with a gel documentation system (LAS-3000 Fujifilm). The levels of phosphorylated proteins (p-p38, pERK1/2, and pRSK) were standardized with relative levels of their total amount. COX-2 and NF-kB were standardized with respect to GAPDH levels and all was expressed as a percentage of controls. We used the following antibodies for the experiments: anti-COX-2 (1:1000 dilution; Merck Millipore, Darmstadt, GER), NF-κB p65 (1:1000 dilution), phospho-ERK1/2 (1:1000 dilution), phospho-p38 (1:1000 dilution), phospho-RSK (1:1000 dilution; Cell signaling, Danvers, EUA), total ERK1/2 (1:1000 dilution; Aviva Systems Biology, San Diego, USA), p38 (1:1000 dilution; Santa Cruz Biotechnology, Santa Cruz, USA), RSK (1:1000 dilution; Cell Signaling Technology), and anti-GAPDH (1:3000 dilution; Sigma-Aldrich).

### Quantitative real-time polymerase chain reaction (PCR) analysis

Macrophages were prepared and subjected to RNA isolation using TRIsure Reagent (Bioline), followed by reverse transcription to cDNA. cDNA was synthesized using a cDNA reverse transcription kit. Quantitative PCR amplification reactions were performed with TaqMan® PCR Master Mix (Invitrogen Life Technologies, Massachusetts, USA) according to the manufacturer’s instructions. Quantitative PCR amplification reactions were performed in the 7500 Fast Real-Time PCR System (Applied Biosystems). The following pre-designed TaqMan assays were used: GAPDH (Mm99999915_g1), IL-1β (Mm00434228_m1), IL-6 (Mm00446190_m1), IL-10 (Mm00439616_m1), and COX-2 (Mm00478377_g1). Values were normalized to GAPDH expression.

### Cell culture and transfection

The mouse COX-2 promoter-luciferase construct pCOX-511, NF-κB promoter-luciferase construct pNFKB-Luc^+^ (Stratagene), and pSV40-β-galactosidase control vector (Promega) were generous gifts from Dr. Francesc Ventura (Universitat de Barcelona). Cells were transfected using Lipofectamine LTX (Invitrogen), and after 6 h the treated group was co-cultivated with MSCs. The next day, cells were serum starved before reaching confluence and treated with LPS (1 μg/mL; Sigma-Aldrich) for 24 and 48 h. Luciferase activities were quantified using the luciferase assay system (Promega, Madison, WI, USA). Luciferase values were normalized using β-galactosidase, and activity measured with the Luminescent β-Galactosidase Detection Kit II (CLONTECH, Palo Alto, CA, USA).

### Statistical analysis

The data were analyzed by one-way analysis of variance (ANOVA) or Student’s *t* test. For comparison of significance, Tukey’s test was used as a post-hoc test according to the statistical program GraphPad Prism. Quantitative data are presented as means ± SEM. Differences were considered significant at *p* < 0.05.

## Results

### MSC administration ameliorates inflammation on sepsis in the lung

**Fig. 1:**
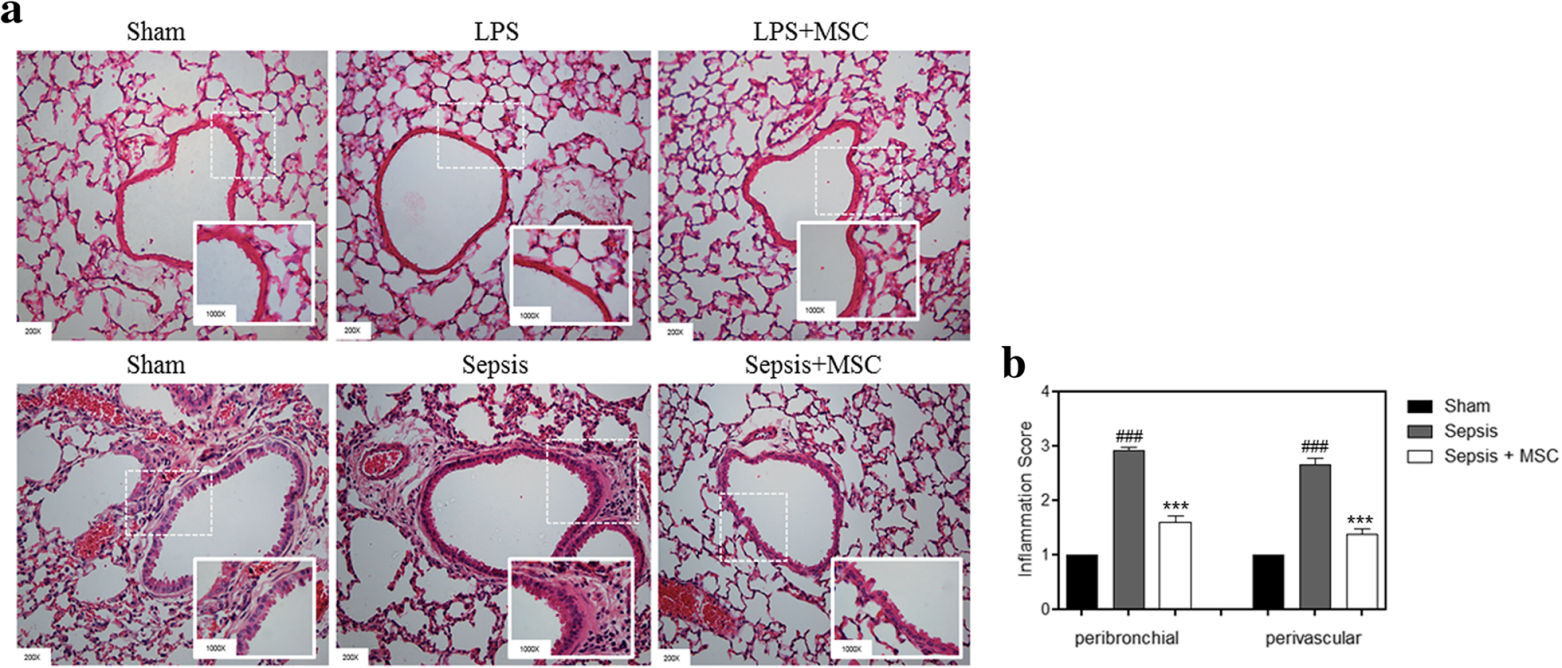
Mice treated with MSCs showed a reduced inflammation score in the lung. **a** Lung sections were subjected to H&E staining. **b** The inflammatory cells that infiltrated into the peribronchial and perivascular lung tissues in the sepsis group were ameliorated after MSC administration (×200 magnification). Data represent the mean ± SEM, *n* = 5. ^###^ *p* < 0.001, sham versus sepsis group; ***p < 0.001, sepsis versus sepsis + MSC group. *LPS* lipopolysaccharide; *MSC* mesenchymal stem cell

As a first step, we used two models of sepsis induction in mice to verify the ability of MSCs to decrease pulmonary inflammation. When we performed H&E staining 12 h after sepsis induction, it was observed that induction using only LPS was not able to cause inflammation in the lung. In contrast, sepsis induction performed with the capsule model, previously standardized by our laboratory, was able to cause an increase in lung cell infiltrates (Fig. 1a).

We calculated the score for inflammatory lung tissue using the software Image-Pro Plus (Medical Cybernetics). It was possible to evaluate in the septic group that there was an increase in the inflammatory score, both peribronchial and perivascular, when compared with the sham group. The MSC-treated group had a reduced score when compared with the septic group, demonstrating the ability of MSCs to decrease inflammation in the lung tissue (Fig. 1b).

**Fig. 2:**
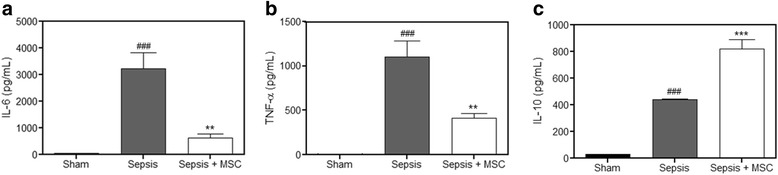
Mice treated with MSCs show reduced inflammatory markers. The pro-inflammatory factors tumor necrosis factor-alpha (*TNF-α*) and interleukin-6 (*IL-6*), and the anti-inflammatory factor IL-10 in the serum were detected by luminescence. The serum showed a significant increase in IL-6 (**a**) and TNF-α (**b**) in the sepsis group compared with the levels in the sham group. A significant decrease in TNF-α and IL-6 was observed in the sepsis + mesenchymal stem cell (*MSC*) group compared with the levels in the sepsis group. Furthermore, the serum showed a significant increase in IL-10 (**c**) in the sepsis group compared with the levels in the sham group, and a greater increase in the sepsis + MSC group when compared with the septic group. Data represent the mean ± SEM, *n* = 10. ^###^ *p* < 0.001, versus sham group; **p < 0.01, ***p < 0.001, versus sepsis group

In previous studies from our laboratory, using this same model, we observed a decreased production of inflammatory cytokines [11]. In the present study, we measured the concentration of two inflammatory markers, IL-6 and TNF-α, and one anti-inflammatory marker, IL-10, in serum. IL-6 and TNF-α were significantly increased in the sepsis group compared with the sham group, and this increase was reduced in the sepsis group treated with MSCs (Fig. 2a and b). Furthermore, the group that received MSCs had a greater increase in IL-10 than the untreated sepsis group (Fig. 2c). These results demonstrate the anti-inflammatory potential of these cells.

### MSCs attenuate COX-2 and NF-κB expression in lung tissue

**Fig. 3:**
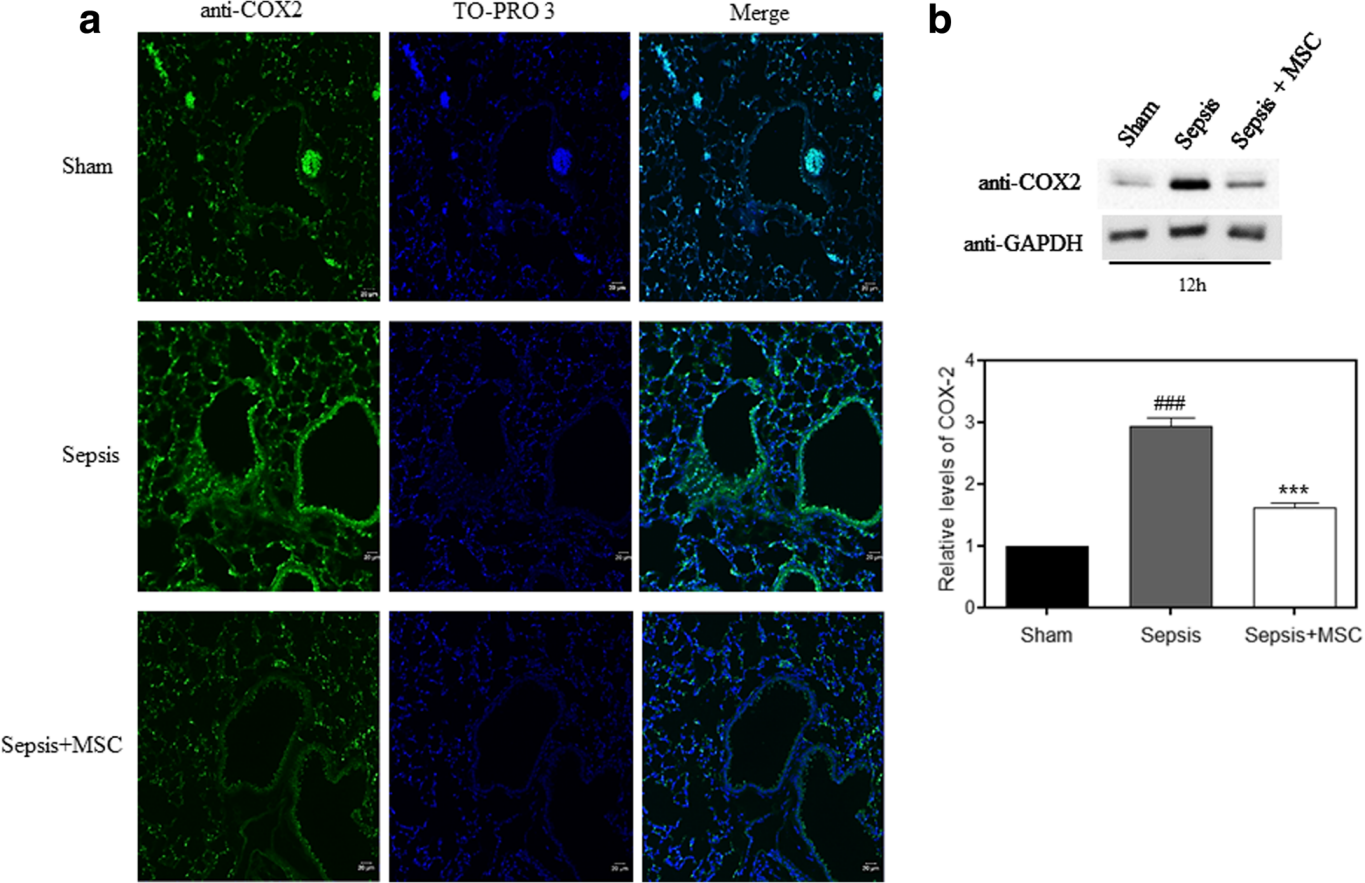
MSC administration reduced elevated COX-2 in lung tissue. **a** Lung sections were prepared, and immunofluorescence was used to assess the expression of cyclooxygenase-2 (*COX-2*; *green*) using a fluorescence microscopy. TO-PRO 3-staining nuclei (*blue*) were also shown. Images show representative data from one of four individual experiments. The COX-2 levels in lung tissue were measured by immunohistochemistry assay. There was a significant increase in COX-2 protein in the sepsis group compared with the sham group. COX-2 expression was decreased in the sepsis + mesenchymal stem cell (*MSC*) group compared with the LPS group (*n* = 5 per group, ×100 magnification). **b** Lung tissues were scraped into a homogenate and analyzed by Western blotting for the expression of COX-2. COX-2 expression was increased in the sepsis group and decreased after MSC administration. Data represent the mean ± SEM, *n* = 5. ^###^ *p* < 0.001, versus the sham group; ***p < 0.001, versus the sepsis group

COX-2 is overexpressed in response to inflammatory inducers and this increase is responsible for the production of various factors involved in the inflammatory process [26]. Thus, we investigated whether MSCs during sepsis were able to decrease the expression of COX-2 in lung tissue. First, we performed immunofluorescence staining and made a comparison between the groups. We saw an increase in the sepsis group compared with the control group. However, this increase was not observed in the sepsis group that received treatment with MSCs (Fig. 3a). To confirm that COX-2 expression was really reduced in the group treated with MSCs, we performed a Western blot and again observed a decrease in the treated group when compared with the untreated sepsis group (Fig. 3b).

**Fig. 4:**
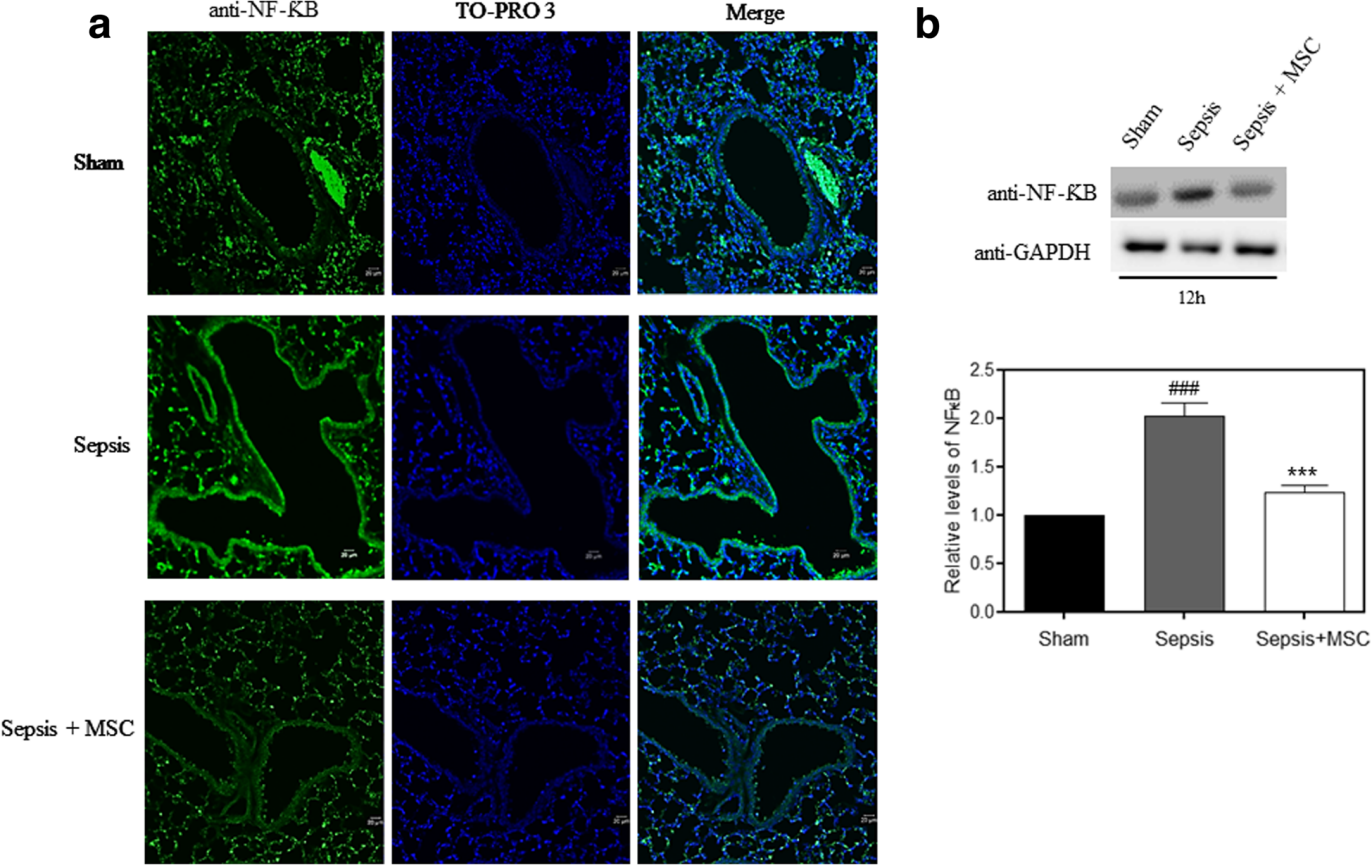
MSC administration reduced elevated NF-κB in lung tissue. **a** Lung sections were prepared, and immunofluorescence was used to assess the expression of NF-κB (*green*) using a fluorescence microscopy. TO-PRO 3-staining nuclei (*blue*) were also shown. Images show representative data from one of four individual experiments. The NF-κB levels in lung tissue were measured by immunohistochemistry assay. There was a significant increase in NF-κB protein in the sepsis group compared with the sham group. NF-κB expression was decreased in the sepsis + mesenchymal stem cell (*MSC*) group compared with the LPS group (*n* = 5 per group, ×100 magnification). **b** Lung tissues were scraped into a homogenate and analyzed by Western blotting for the expression of NF-κB. NF-κB expression was increased in the sepsis group and decreased after MSC administration. Data represent the mean ± SEM, *n* = 5. ^###^ *p* < 0.001, versus the sham group; ***p < 0.001, versus the sepsis group

We also investigated if there was a reduction in the expression of NF-κB, which is a transcriptional regulator and induces the expression of several genes including COX-2. When we performed immunofluorescence staining, we observed that the sepsis group had increased NF-κB compared with the control group, and that this increase was reduced on treatment with MSCs (Fig. 4a). We also conducted Western blotting to confirm this decrease. The increase in NF-κB expression in the MSC-treated group is significantly reduced when compared to the sepsis group (Fig. 4b).

### LPS-induced pERK, pRSK, p-p38, and NF-κB upregulation in the macrophage cell lines

Macrophages are directly involved in inflammation and are responsible for the release of several cytokines and increased COX-2 expression [27]. To investigate the possible mechanism by which MSCs can reduce lung inflammation during sepsis, we performed in vitro experiments using a cell line of pulmonary macrophages (RAW 264.7) stimulated with LPS. We know that the principal route of activation of COX-2 production and other inflammatory cytokines such as TNF-α and IL-1 occurs via the MAPK pathways, so we attempted to verify that this could be a potential mechanism of action of the MSCs.

**Fig. 5:**
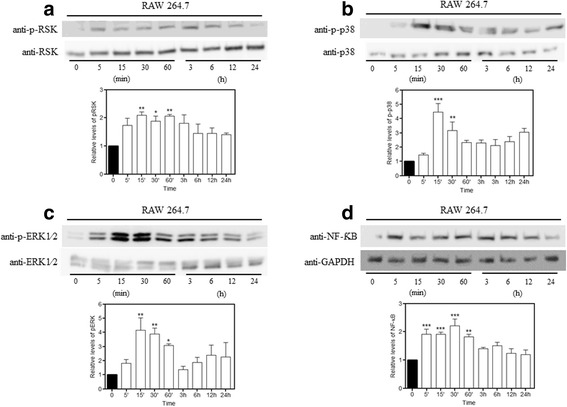
LPS-induced MAPK pathway activation. We performed a time-course experiment to evaluate the best times for the MAPK pathway activation. Macrophages were cultured with or without LPS (1 μg/mL) for 5, 15, 30, and 60 min and 3, 6, 12, and 24 h. Protein expression was evaluated using Western blot analysis. LPS induced a significant increase in the expression of (**a**) p-RSK, (**b**) p-p38, (**c**) p-ERK, and (**d**) NF-κB at different times. Data represent the mean ± SEM, *n* = 5. *p < 0.05, **p < 0.01, ***p < 0.001, versus sham

First, we performed a time-course experiment to evaluate the best activation times of these proteins by Western blot. We tested the phosphorylation of p38, ERK1/2, and RSK, which is a substrate of ERK, and the NFκB expression. The best activation times were concentrated between 5 min and 24 h, so we chose three times—15, 30, and 60 min—for the following experiments (Fig. 5).

### LPS-induced pERK, pRSK, p-p38, and NF-kB expression is reduced in macrophages co-cultured with MSCs

**Fig. 6:**
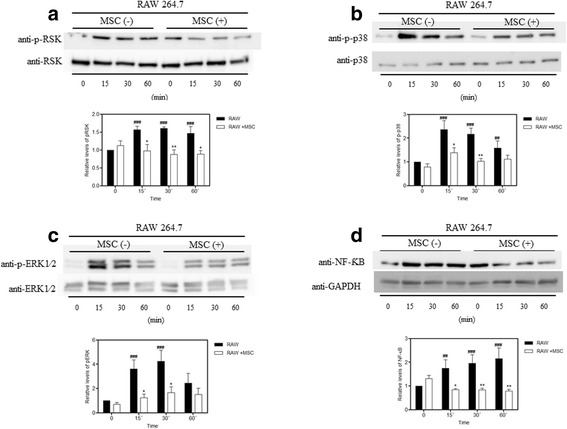
Co-cultivation with MSCs inhibited LPS-induced phosphorylation of RSK, p38, ERK, and NF-κB expression in macrophages. Macrophages were treated with LPS (1 μg/mL) for 15, 30, and 60 min. The MAPK phosphorylation and NF-κB expression were evaluated by Western blot analysis. **a** RSK was phosphorylated at 15, 30, and 60 min in response to LPS. The co-culture with mesenchymal stem cells (*MSCs*) inhibited the activation-associated phosphorylation of RSK at all times. **b** p38 phosphorylation was increased in the macrophages treated with LPS after 15, 30, and 60 min, and decreased upon co-cultivation with MSCs at 15 and 30 min. **c** ERK was phosphorylated after 15 and 30 min in response to LPS stimulation and this phosphorylation could be inhibited by co-cultivation with MSCs. Blots are representative of at least four separate experiments. **d** NF-κB expression was increased in the LPS group after 15, 30, and 60 min. This increase was inhibited by MSCs at all times. Data represent the mean ± SEM, *n* = 5. ^##^ *p* < 0.01, ^###^ *p* < 0.001, versus corresponding untreated controls; *p < 0.05, **p < 0.01, versus corresponding RAW counterparts

To investigate if the MSCs were able to reduce the activation induced by LPS, we co-cultivated macrophages with MSCs. Macrophages were co-cultured for 24 h with the MSCs and, afterwards, were treated with LPS according to the predefined times. NF-κB and p-RSK were reduced in the RAW + MSC group when compared with the group exposed to LPS without co-cultivation at all times (Fig. 6a and d). Macrophages co-cultured with MSCs had decreased phosphorylation of p-p38 and p-ERK at 15 and 30 min compared to the RAW group stimulated with LPS (Fig. 6b and c).

**Fig. 7:**
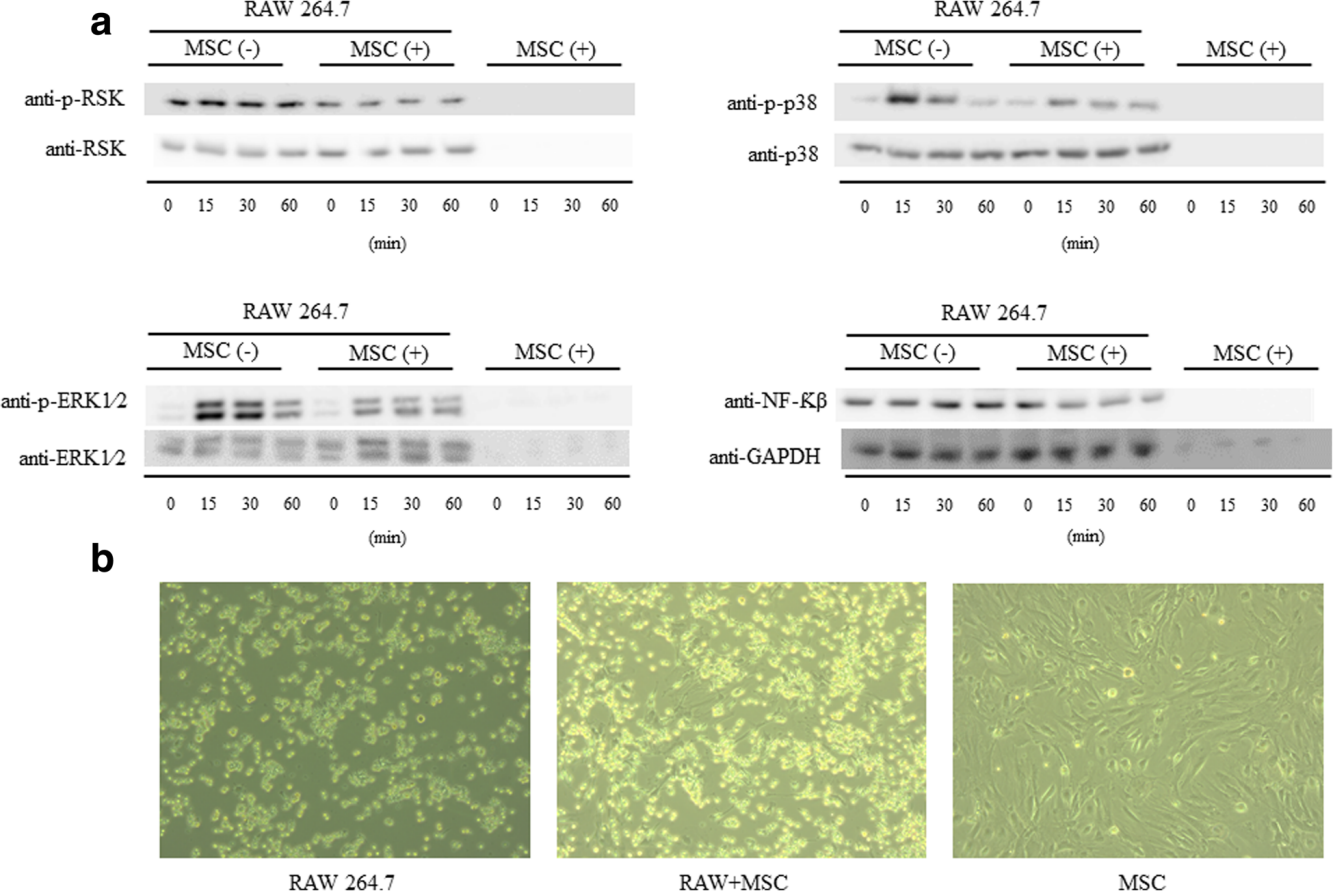
An MSC fraction directly co-cultivated without macrophages does not influence the results found in the Western blot. **a** We analyzed whether the protein expression of MAPKs and NF-κB found in Western blot could be due to the mesenchymal stem cells (*MSCs*). We did not observe any expression from the MSCs alone. **b** Representative image of co-cultivation between macrophages and MSCs

We included a group with only stem cells (without co-culture with macrophages) to demonstrate that there is no influence or background expression from the MSCs, and the results show that the expression of proteins studied comes only from macrophages (Fig. 7). We performed these Western blot experiments using proportionally 10% of the amount of protein used in the co-cultivated group, since in the RAW + MSC group the cells are plated proportionally at 10:1.

**Fig. 8:**
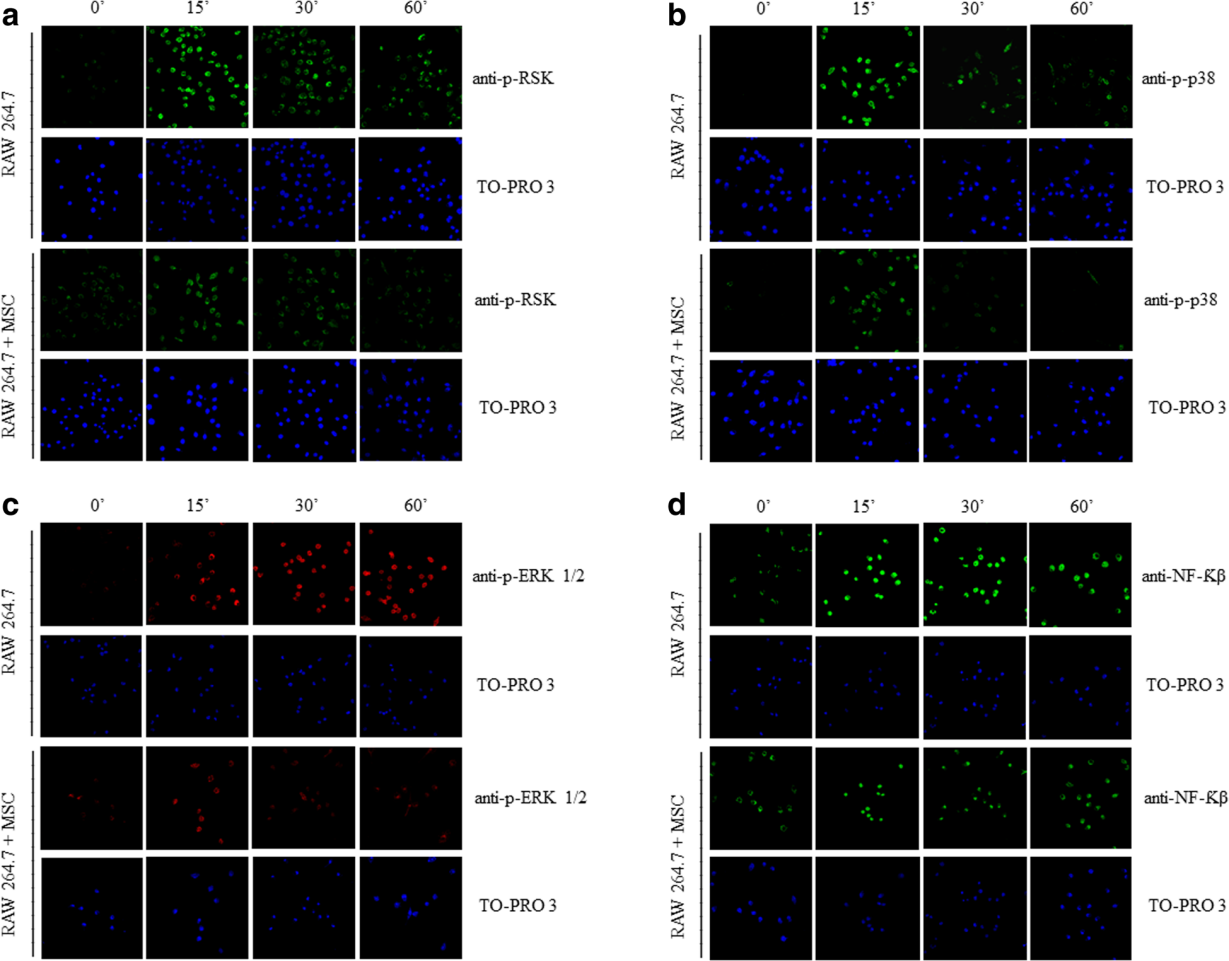
Immunohistochemistry analysis of the expression of pRSK, p-p38, p-ERK, and NF-κB in macrophages. Macrophages were prepared and immunofluorescence was used to assess the expression of (**a**) pRSK, (**b**) p-p38, (**c**) p-ERK, and (**d**) NFκB using fluorescence microscopy. TO-PRO-3-staining nuclei (*blue*) are also shown. Images show representative data from one of four individual experiments. pRSK, p-p38, p-ERK, and NF-κB expression increased in the macrophages treated with LPS when compared with the untreated group. This increase was reduced in the group co-cultured with mesenchymal stem cells (*MSCs*). *n* = 5, ×20 magnification

To confirm our findings by Western blot, we performed immunofluorescence staining in all groups for p-p38, p-ERK1/2, p-RSK, and NF-κB. Our results showed the same profile found from the Western blot. It was shown that co-cultivation with MSCs inhibited NF-κB expression and the phosphorylation of p-38, ERK1/2, and RSK when compared with the group that had only induction with LPS. All these results demonstrate the ability of these cells to prevent the activation of the MAPK pathway that is essential for inflammation (Fig. 8).

### Macrophage cell lines co-cultured with MSCs prevent LPS-induced COX-2 and NF-kB expression over the long-term

**Fig. 9:**
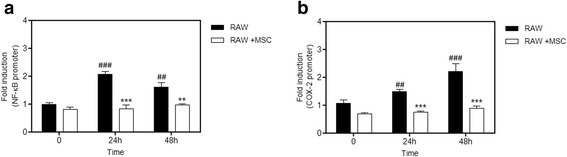
Induction of COX 2 and NF-κB expression over the long term is reduced by co-cultivation with MSCs. Macrophages were transfected with plasmids as described in the Methods section to evaluate the expression of COX 2 and NF-κB. Macrophages were then co-cultivated or not with mesenchymal stem cells (*MSCs*), and stimulated with LPS. The induction fold was determined and the luciferase activity was normalized to the β-galactosidase activity. **a** NF-κB expression increased in the macrophages treated with LPS when compared with the untreated group at 24 and 48 h. This increase is reduced in the group co-cultured with MSCs. **b** There was a significant increase in COX-2 protein expression in the group treated with LPS when compared with the untreated group at 24 and 48 h. This increase was inhibited by co-cultivation with MSCs. Data represent the mean ± SEM, *n* = 5. ^##^ *p* < 0.01, ^###^ *p* < 0.001, versus corresponding untreated controls; **p < 0.01, ***p < 0.001, versus corresponding RAW counterparts

We wanted to know if the inhibition of phosphorylation in the MAPK pathways caused by MSCs could affect the expression of COX-2 and NF-κB over the long term, maintaining the anti-inflammatory effect. Thus, we transfected macrophages with a COX-2 promoter-luciferase construct and NF-κB promoter-luciferase construct separately. According to the analysis of luciferase activity, we observed a significant increase at 24 h and 48 h in the group treated with LPS compared with the control group for both COX-2 and NF-κB promoter activity. This increase was not observed in the group co-cultured with MSCs. This result indicates that a possible initial inhibition of phosphorylation of MAPKs by MSCs can lead to inhibition of COX-2 and NF-κB over the long term, and demonstrates the ongoing anti-inflammatory potential of the MSCs (Fig. 9).

### MSCs attenuate LPS-induced IL-1, IL-6, and COX-2, and promote IL-10 expression in macrophages

**Fig. 10:**
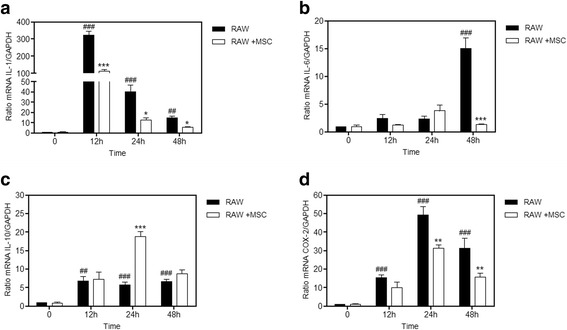
MSCs attenuated LPS-induced IL-1, COX-2, and IL-6 production, and promoted IL-10 production in macrophages. Macrophages were cultured with or without mesenchymal stem cells (*MSCs*) with LPS (1 μg/mL) for 12, 24, and 48 h, and were subjected to real-time PCR analysis. LPS significantly increased the (**a**) interleukin (*IL*)-1 (12, 24, and 48 h), (**b**) IL-6 (48 h), and (**d**) cyclooxygenase-2 (*COX-2*) (12, 24, and 48 h) when compared with the control group. In the group co-cultivated with MSCs, the mRNA levels of IL-1, IL-6, and COX-2 decreased significantly when compared with the respective LPS group, while (**c**) the IL-10 mRNA levels increased significantly at 24 h compared with the LPS group. Data represent the mean ± SEM, *n* = 5. ^##^ *p* < 0.01, ^###^ *p* < 0.001, versus corresponding untreated controls; *p < 0.05, **p < 0.01, ***p < 0.001, versus corresponding RAW counterparts

The macrophages exposed to LPS for 24 h with and without co-culture were collected for analysis by quantitative PCR of the mRNA levels of IL-1, IL-6, IL-10, and COX-2. The group treated with LPS had a significant increase in IL-1 at 12, 24, and 48 h. At 48 h, we saw an IL-6 increase in the sepsis group when compared with the control group. In all cases, co-culture with MSCs was able to significantly reduce this increase (Fig. 10a and b). The levels of the anti-inflammatory cytokine IL-10 increase in the groups treated with LPS at 12, 24, and 48 h, and MSCs provoked a significant increase when compared to the group treated with LPS at 24 h (Fig. 10c). COX-2 levels are increased in the LPS groups at all times, and the MSCs induced a significant reduction at 24 and 48 h (Fig. 10d).

### MAPK p38 and ERK signaling is required for MSC-mediated LPS-induced COX-2 and NF-κB expression in macrophages

**Fig. 11:**
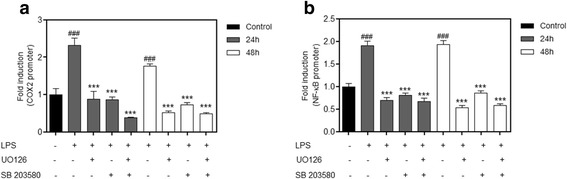
Inhibition of p38 and ERK has the same effect as that caused by MSCs co-cultured with macrophages stimulated with LPS. Macrophages were transfected with plasmids as described in the Methods section to evaluate the expression of cyclooxygenase-2 (*COX-2*) and NF-κB. Macrophages were treated with the p38 inhibitor SB203580 and the ERK inhibitor U0126 and stimulated with lipopolysaccharide (*LPS*). The induction fold was determined and the luciferase activity was normalized to the β-galactosidase activity. LPS-induced (**a**) COX-2 and (**b**) NF-κB upregulation was reduced by the p38 inhibitor (SB203580) and the ERK inhibitor (U0126) at 24 and 48 h. We did not observe any synergistic effect when both inhibitors were administered at the same time. Data represent the mean ± SEM, *n* = 5. ^###^ *p* < 0.001, versus corresponding untreated controls; ***p < 0.001, versus the LPS-only group

Subsequently, we attempted to prove that the activation of p38 and ERK has an important role in the upregulation of COX-2 and NFκB, and the effects seen on treatment with MSCs possibly occur through this pathway. We measured the expression of COX-2 and NFκB using a specific inhibitor for p38, SB203580, and another for ERK, U0126. Macrophages previously transfected with the reporter genes were treated with inhibitors and subsequently subjected to LPS for 24 and 48 h. We observed a decrease in the promoter activity of COX-2 and NF-κB with both inhibitors when compared with the group treated with LPS (Fig. 11). We also measured a group using both inhibitors at the same time to verify any synergistic effect, but none was found. These data suggest that MSCs can decrease COX-2 and NF-κB expression via the MAPK pathway, p38 and ERK, thereby modulating the inflammation.

## Discussion

In this study we evaluated the effect of MSC therapy in a sepsis model and its possible mechanism of action in pulmonary macrophages. Our study suggests that MSC injection into a sepsis model can improve the inflammation acting via inhibition of the MAPK pathway by generating decreased production of inflammatory mediators.

Sepsis is defined by a generalized inflammation due to a host immune disorder, dysregulation of the clotting cascade, and endothelial dysfunction in response to the invading pathogenic agents [28]. This can progress to septic shock, generating significant dysfunction of multiple organs and potentially resulting in death. Over the past years, several studies have shown the ability of MSCs to attenuate the dysfunction of organs and improve survival in several models of sepsis in animals, suggesting its potential use for treatment of patients with sepsis [11, 16, 29, 30]. MSCs have an intrinsic capacity to migrate to injured tissues, such as the lung, myocardium, brain, liver, and kidney, and can improve the lesion by reducing both local and systemic inflammation via a decrease in the production of pro-inflammatory cytokines and an increase in production of anti-inflammatory cytokines [31, 32]. In this study, we demonstrated a possible immunomodulatory mechanism of MSCs through this process.

First, we evaluated two models in vivo to identify which would have a greater inflammatory score and which we should use to study the possible mechanism of action of MSCs on the inflammatory response. Our evidence showed that the severe sepsis model developed by our laboratory was a better way, and we thus performed in vivo experiments with this model.

Sepsis causes multiple organ dysfunction, including the cardiovascular system, liver, kidney, and lung. Fifty percent of patients with severe sepsis will develop acute lung injury (ALI) and it is generally considered that damage to the lung is one of the most severe outcomes of sepsis [33]. It was observed that intravenous MSC administration attenuated the score of lung inflammation in our study.

MSCs display some immunosuppressive characteristics that may be quite important in sepsis [34, 35]. Soluble paracrine signals from MSCs have been shown to downregulate inflammatory cytokine production by macrophages and inhibit neutrophil chemotaxis [36]. When we analyzed the interleukin levels in the serum of the animals, we observed a reduction in inflammatory cytokines IL-6 and TNF-α, and an increase in the production of the anti-inflammatory IL-10, corroborating previous studies [11, 18].

Sepsis is a systemic inflammatory response against bacterial agents. This inflammatory response is characterized by increased inflammatory cytokines TNF- α, IL-1, and IL-6, and anti-inflammatory cytokines, especially IL-10 [37]. Activation of MSCs by pro-inflammatory factors triggers two paracrine negative feedback loops that can reduce inflammation. The first loop involves PGE2, as demonstrated in a study by Németh et al. [18] using a model of sepsis in mice induced by cecal ligation and puncture. In the same study, in in-vitro experiments, MSCs were shown to secrete PGE2 in response to endotoxin (LPS). PGE2 altered the activity of tissue-resident macrophages with a pro-inflammatory response and stimulated the secretion of an array of anti-inflammatory mediators such as IL-10 and IL-1 receptor antagonist, reducing the systemic effects of sepsis and thus improving survival [18]. However, although these results demonstrate that MSCs reprogram macrophages, how this occurs remained uncertain.

COX-2 is transiently induced after mitogenic or inflammatory stimuli, implicating it in the production of prostaglandins involved in inflammatory responses [38]. Our in vivo results showed that administration of MSCs could decrease COX-2 production following sepsis induction. This led us to seek a possible mechanism of action in vivo in macrophages to justify this inhibition found in the in vivo model.

The pathway that usually regulates COX-2 expression in inflammation involves a family of highly conserved intracellular signaling molecules, the MAPKs [39]. Generally, ERK1/2 responds to mitogens and growth factors that regulate cell proliferation and differentiation, whereas p38 is activated by environmental stresses, such as UV radiation, heat shock, and pro-inflammatory cytokines such as IL-1 and TNF-α [21].

We selected macrophages, which are the predominant immune effector cells, to act as mediators of the inflammatory response and which contribute to both the initiation and resolution of inflammation [40]. First, we evaluated if LPS was effective in activating the MAPK pathway, and we chose short time points since we saw with those a better activation response. When we co-cultivated macrophages with MSCs we observed a significant reduction in phosphorylation and that a possible route of action of the MSCs was via the MAPK pathway. This phosphorylation-inhibition mechanism of MSCs on the MAPK pathway is poorly described. Recent studies using a model of cigarette smoke induction showed results similar to the findings in our research [39].

Recent studies have shown that MAPKs can bind to and stimulate kinase targets, translocate to the nucleus, and activate NF-κB transcription [41]. The ability of MSCs to inhibit the MAPK pathway by co-cultivation is essential for inhibiting the production of NFκB and subsequently the production of other inflammatory mediators. This factor regulated inflammatory cytokines, mainly IL-1, IL-6, and TNF-α. As is seen in the in vivo experiments, NF-κB expression in vitro is also reduced.

Continuing this work, we attempted to investigate if this inhibition profile of COX-2 and NF-κB remained over a longer period of time, and if somehow it could still have an effect on the production and release of pro- and anti-inflammatory factors derived from macrophages. When we transfected the macrophages with a reporter gene for COX-2 and NF-κB we observed that this decrease was maintained in the group co-cultured with MSCs. In addition, mRNA levels of pro-inflammatory cytokines IL-1 and IL-6 were higher than in the group co-cultured with MSCs; however, the increase in the anti-inflammatory IL-10 was significantly higher in the co-cultured cells. This shows that the reduction of inflammatory mediators and reprogramming of macrophages caused by MSCs was possibly through the MAPK pathway.

Furthermore, we wanted to show that the action of MSCs was via the MAPK pathway. Thus, we used two specific inhibitors, one for p38 and another for ERK. When we performed an analysis of COX-2 and NF-κB levels, the results showed that both inhibitors were able to inhibit the production of both NF-κB and COX-2, showing a similar profile to that found when macrophages were co-cultured with MSCs. Altogether, these results suggest that the reduced production of inflammatory mediators caused by MSCs comes from their ability to inhibit the MAPK pathway which would cause a decrease in production of COX-2, NF-κB, and other inflammatory mediators during sepsis.

## Conclusions

Our study was able to show that the immunomodulatory effect of MSCs is directly related to their ability to inhibit the activation of MAPKs such as ERK, RSK, or p38, causing a decrease in the production of COX-2 and NF-κB, and therefore inflammatory cytokines. The way that MSCs act and are able to influence the host response is essential for an understanding in the search for new alternative treatments for sepsis.

## Abbreviations

COX-2: Cyclooxygenase-2
DMEM: Dulbecco’s modified Eagle’s medium
ERK: Extracellular signal-regulated kinase
FBS: Fetal bovine serum
H&E: Hematoxylin and eosin
IL: Interleukin
JNK: c-JUN N-terminal kinases
LPS: Lipopolysaccharide
MAPK: Mitogen-activated protein kinase
MSC: Mesenchymal stem cell
NF-κB: Nuclear factor kappa B
PBS: Phosphate-buffered saline
PCR: Polymerase chain reaction
PGE2: Prostaglandin E2
TNF: Tumor necrosis factor
